# Do emotions influence safe browsing? Toward an electroencephalography marker of affective responses to cybersecurity notifications

**DOI:** 10.3389/fnins.2022.922960

**Published:** 2022-07-14

**Authors:** Colin D. Conrad, Jasmine R. Aziz, Jonathon M. Henneberry, Aaron J. Newman

**Affiliations:** ^1^School of Information Management, Dalhousie University, Halifax, NS, Canada; ^2^Department of Psychology and Neuroscience, Dalhousie University, Halifax, NS, Canada

**Keywords:** security warnings, affective processing, decision making, electroencephalography (EEG), event-related potentials (ERP), late positive potential (LPP)

## Abstract

Cybersecurity notifications play an important role in encouraging users to use computers safely. Emotional reactions to such notifications are known to positively influence users’ adherence to these notifications, though it is challenging for researchers to identify and quantify users’ emotional reactions. In this study, we explored electroencephalography (EEG) signals that were elicited by the presentation of various emotionally charged image stimuli provided by the International Affective Picture System (IAPS) and compared signals to those elicited by images of cybersecurity notifications and other computer-related stimuli. Participants provided behavioral assessments of valence and arousal elicited by the images which were used to cross-reference the results. We found that EEG amplitudes corresponding to the late positive potential (LPP) were elevated in reaction to images of cybersecurity notifications as well as IAPS images known to elicit strong positive and negative valence, when compared to neutral valence or other computer-related stimuli. These findings suggest that the LPP may account for emotional deliberation about cybersecurity notifications, which could be a useful measure when conducting future studies into the role such emotional reactions play in encouraging safe computer behavior.

## Introduction

Users of information technologies are often presented with complex decisions and utilize multiple cognitive processes to arrive at an action (Dimoka et al., 2011). In order to respond to computer security notifications, for instance, users must weigh utilitarian goals with their need to protect themselves. When presented with a decision about whether to proceed to a website or return to a safer page, a user may weigh their options and consider the context that they find themselves in. However, they may also simply ignore the message, perhaps because these messages have become mundane.

Researchers have investigated factors that facilitate safe computer behavior, and for good reason. Computer security has recently been recognized as a major topic in computing, in part because of the growing importance of information technology infrastructure (Schaumont and Montuschi, 2021). From the perspective of human decisions, much of the past research on this topic had concerned users’ habituation to notifications and the impact of various emotional factors, such as fear of negative consequences (Anderson et al., 2016). These considerations have been intertwined with theories of motivation. The influential protection motivation theory proposed by Rogers (1985) posits that there are four perceptions that could influence a users’ response to a threat: susceptibility, severity, response efficacy, and personal ability to respond. Emotional reactions elicited by security notifications could change a user’s perception of susceptibility and risk severity, ultimately leading individual to make safer decisions (Hanus and Wu, 2016). Computer security designers could utilize insights about a notification’s ability to shape risk perceptions to improve future notifications (van Bavel et al., 2019).

A major challenge with Rogers’ model of motivation is that it can be difficult to disentangle the elements of an individual’s decision-making process that lead to persuasion. For example, provocative or emotionally charged notifications may initially draw a user’s attention, though the notifications might eventually seem unrealistic or untrustworthy if a user is repeatedly presented with such messages, leading to habituation (Vance et al., 2014). Past studies that have utilized self-report and behavioral measures provide evidence which suggests that perceived severity of computer threats (Johnston and Warkentin, 2010) and perceived security risk (Guo et al., 2011) influence attitudes toward notifications. However, these reports do not distinguish specific cognitive factors that may influence an individual’s decision to take risky actions (Samtani et al., 2020).

Electroencephalography (EEG) has potential to improve upon such measures by providing insight into the neural mechanisms that are engaged when viewing cybersecurity notifications. EEG has also been employed in the study of cybersecurity notifications before. Vance et al. (2014) observed the P300 event-related potential (ERP) in the context of a gambling risk task and found that P300 responses to risk-related stimuli were a strong predictor of a participant’s propensity to disregard security responses, even when compared to common questionnaire measures. The P300 component is elicited when individuals attend to a task-related stimulus and is likely amplified by the activation of working memory or related executive processes (Vogel and Luck, 2002; Luck, 2014). It follows that individuals who spend executive resources on risk deliberation would be more likely to respond to cybersecurity notifications. The results of that study suggest that neurophysiological measures can contribute to the improvement of cybersecurity decision models and give insight into antecedents of safe computer behavior.

Building on these findings, we propose investigation of another ERP component which may give additional insight into risk deliberations. The late positive potential (LPP) is a signal that is closely related to the P300 component and reflects task relevance to an emotion-inducing stimulus (Luck, 2014). The amplitude of the LPP can be modulated by the salience of a stimulus and by an individual’s emotional reaction to stimuli (Hajcak and Olvet, 2008; Brown et al., 2012; Bublatzky and Schupp, 2012; Minnix et al., 2013). By measuring the P300 and LPP, we can potentially identify a method for indexing both an individuals’ perceived task relevance, and their emotional response to various security notification stimuli.

An ERP method for user emotion detection has some advantages over other methods often employed by user experience researchers such as machine learning facial recognition. Facial recognition approaches employ computer vision and machine learning to classify segments of a user’s facial images to infer a user’s emotional state (Garcia-Garcia et al., 2017). While these approaches are easy for researchers to implement, they are also susceptible to bias, as users may either advertently or inadvertently change their facial expressions to mimic a different emotion than what they are currently experiencing (Landowska and Miler, 2016). Furthermore, there is evidence that these techniques have limited accuracy, as even premium facial recognition solutions may even misclassify emotional states up to 20% of the time (Skiendziel et al., 2019). An ERP approach to emotion recognition could thus complement other methods by offering temporal insights into the onset of the emotional reaction and help improve the reliability of emotion detection in user experience research.

We thus developed an experiment that compared P300 and LPP responses elicited by pictures of various cybersecurity notifications with pictures of non-threatening computer stimuli and pictures from the International Affective Picture System (IAPS; Lang, 2005), a well-studied repository of images that have been indexed for the systematic study of attention and emotion. Though the IAPS is most often used to investigate the specific physiological processes related to emotional processing, it has also been applied in broader contexts such as exploring the emotional effect of art (Gerger et al., 2014) and emotions in virtual reality environments (Marín-Morales et al., 2018). In addition, we cross-referenced our findings with behavioral responses to the Self-Assessment Manikin (SAM), a well-studied affective rating system, which can help establish confidence in the veracity of our neurophysiological measures. The SAM facilitates the measurement of valence and arousal triggered by a stimulus, two dimensions of emotional responses that are relevant to the cybersecurity context. The SAM uses a pictorial scale to allow participants to express valence as a continuum between unpleasantness and pleasantness, as well as arousal as a continuum between low to high intensity. By cross-referencing the various ERP and behavioral measures with the IAPS images, we can establish the viability of those indicators as predictors of perceived task-relevance or emotional processing in the context of cybersecurity notifications (Bradley and Lang, 1994; Vance et al., 2014, 2018).

We hypothesized that P300 amplitude elicited by the images would be influenced by the perceived relevance of an image to the task, being amplified for computer-related non-threatening stimuli and cybersecurity warning images. We also predicted that LPP amplitude would be associated with an individuals’ emotional processing of the image, and would be amplified among the cybersecurity, positive valence and negative valence images. The pattern of ERP findings will be interpreted with reference to the self-reported levels of valence and arousal by image condition. In addition to establishing the viability of the measures, these findings would suggest that individuals conduct a degree of emotional processing when presented with images of security notifications.

## Methods

### Participants

Twenty-five university students gave written consent to participate in the experiment. Data from three participants were treated as pilot data and were described in a prior published work-in-progress paper (Conrad et al., 2020). These three participants were omitted from this analysis, while two participants were omitted due to technical challenges during recording, leaving a final sample of twenty participants (15 women and 5 men, aged 18–28 years; *M* = 22.25, *SD* = 3.19). Prospective participants were excluded from the study if they had a condition (e.g., epilepsy or a recent concussion) or were taking medication (e.g., anti-anxiety medication) which could lead to abnormal EEG. Participants were asked to disclose all potential conditions or medications which were in-turn investigated by the team for their effects on EEG using literature search. Participants were also excluded if they had uncorrected vision problems, or were unable to use a keyboard. Participants were informed about the purpose of the study in advance, provided written consent, and were compensated with either course credit or CAD $20 for their time. All procedures adhered to the Canadian Tri-Council Policy Statement on Ethical Conduct for Research Involving Humans 2nd edition, were reviewed, and approved by the Dalhousie University Research Ethics Board.

### Stimuli

Experimental stimuli included 2 categories of computer stimuli (security notification images and neutral computer-related images) and 3 categories of photos from the IAPS database (negative, neutral and positive). Security notification images consisted of computer-based images used by antivirus, web browser and firewall systems (e.g., Chrome, McAfee, Norton), while the computer-related images consisted of non-threatening images typical of a computing environment (e.g., a screenshot of a search engine or Wikipedia). All images were corrected for luminance to control for the effect of luminance on EEG signals (Bradley et al., 2007) and were balanced according to color by ensuring that the number of images with red backgrounds, which are common among cybersecurity notifications, were balanced between the conditions. A collection of both modern and antiquated security notifications were selected to give a greater range of baseline data.

### Self-assessment measures

At the outset of the experiment, participants were asked about their age, gender, perceived skill at using computer systems, years of education and native language, as well as risk questionnaires investigated by Vance et al. (2014). To assess participants’ perceived reaction to the photo stimuli, we used a simplified version of the SAM (Leventon and Bauer, 2016). The manikin was presented 2–3 s following the appearance of the stimulus photos and consisted of two 5-point scales which measure degrees of valence and arousal, respectively. Following the experiment, participants completed a questionnaire about their attitudes toward risk and perception of the impact of computer malware (Vance et al., 2014).

### Procedure

Participants underwent a consent protocol, were fitted with an EEG cap, and then brought to a controlled environment. After participating in an initial questionnaire, participants were asked to attend a series of images and then complete the SAM measure of valance and arousal following each picture. Participants presented with a randomized series of images consisting of IAPS photos, security notifications, and security-unrelated computer phenomena. Each photo was adjusted to 1,280 px by 720 px and presented at the center of a 24-inch monitor for 2 s. Following the SAM ratings, participants had a second break that lasted a random duration between 2 and 3 s during which only a fixation cross was present. Stimuli were delivered using PsychoPy (Pierce, 2009), which was also used to mark the onset of each picture in the EEG recordings *via* transistor-transistor logic (TTL) codes. The stimuli were delivered following a within-subject design, presenting 32 instances of each of the five image conditions. Following the study, participants completed the aforementioned post-questionnaire. Each session took between 60- and 120-min total. Following the session, participants were debriefed and received compensation.

### Data acquisition and processing

Participants were fitted with 32 scalp electrodes (ActiCap, BrainProducts GmbH, Munich, Germany) positioned at standard locations according to the international 10-10 system and referenced to the electrode average. Electrode impedances were kept below 30 kOhm at all channel locations throughout the experiment. EEG data were recorded using an Refa8 amplifier (ANT, Enschende, The Netherlands) at a sampling rate of 512 Hz, were bandpass filtered between 0.01 and 170 Hz, and saved using ANT ASAlab.

Using the EEGlab software, the data were converted from the ASAlab format to EEGlab’s format to facilitate the analysis using open source software (Delorme and Makeig, 2004). Data processing and statistical analysis was conducted in Python using the MNE Python library (Gramfort et al., 2014). Data were filtered using a 0.1–40 Hz bandpass filter and were manually inspected for excessively noisy electrodes, which were removed as needed. The data were segmented into,2200 ms epochs spanning from 200 ms before the stimulus onset, through the 2,000 ms duration of the photo stimuli, as done previously for analysis of the LPP (Hajcak and Olvet, 2008). Epochs were manually inspected and those with excessive noise were removed as needed, following the recommendations outlined by Luck (2014), the MNE library (Gramfort et al., 2014), and the detailed criteria described in our jupyter notebooks, provided as a supplement to this document. Independent component analysis (ICA) was used to remove systematic artifacts from the data, including those created by eye blinks, eye movements, and muscle contractions (Delorme and Makeig, 2004). Following artifact removal data were re-referenced to the TP9 and TP10 electrodes at the mastoids. All data cleaning decisions were recorded using the Jupyter notebook for reproducibility.

### Statistical analysis

Each participant yielded a maximum of 32 epochs for each condition. The dependent EEG measures analyzed were mean amplitude on each trial between 200 and 600 ms (corresponding to the P300 component) as well as mean amplitude between 600 and 1,950 ms following stimulus onset, which corresponds to the window of the LPP component. Statistical analyses were performed on a region of interest which included electrodes Cz, CP1, CP2, and Pz using linear mixed effects (LME) modeling (Davidson, 2009; Tremblay and Newman, 2015). Image condition was treated as a fixed effect and random effects included random intercepts for participants, random slopes for electrode by subject, and random slopes for condition by-subject. The online security warnings condition was selected as the reference level for contrasts the LME analyses. SAM responses were analyzed by ordinal regression using cumulative link models using a cauchit link due to expected observed kurtosis in the responses (Christensen, 2018). Similarly, image condition was treated as an independent variable and the online security warnings condition was selected as the reference level for contrasts.

## Results

**FIGURE 1:**
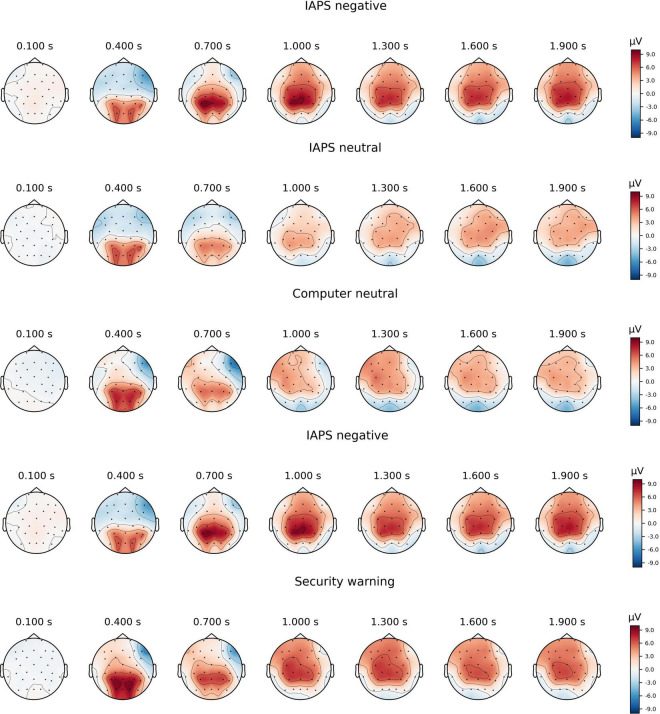
Topographic maps of grand average amplitudes over time between 0 and 1,950 ms at 300 ms intervals for each of the five stimuli conditions.

**FIGURE 2:**
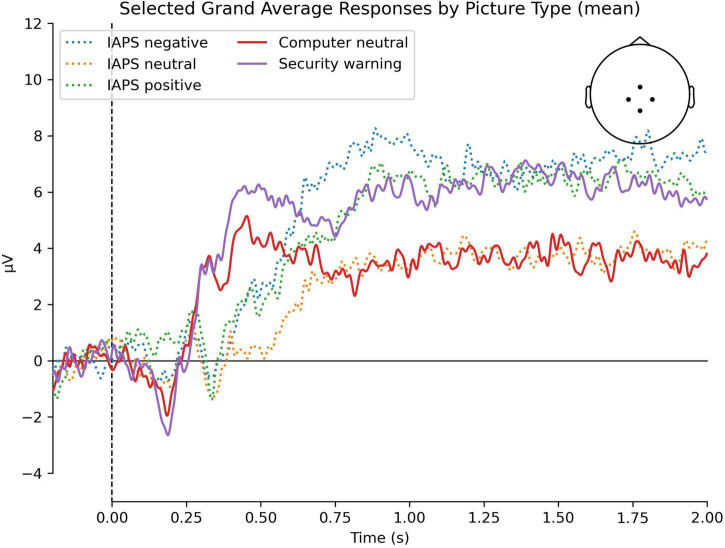
Grand average waveform elicited by the IAPS negative, IAPS neutral, IAPS positive, computer neutral, and security warning stimuli. The image depicts the grand average at channels Cz, CP1, CP2, and Pz.

Figure 1 provides topographic maps of the ERPs over time, while Figure 2 provides a grand average ERP waveform for the region of interest that we analyzed at CPz. Consistent with our prediction of a P300 component, we observed a positive-going peak around 400 ms that was larger for valenced than neutral IAPS stimuli, and larger for security warnings than neutral computer stimuli. Also consistent with our predictions, ERP amplitudes were positive and sustained from 600 ms to the end of the epoch for all conditions, consistent with the LPP.

**FIGURE 3:**
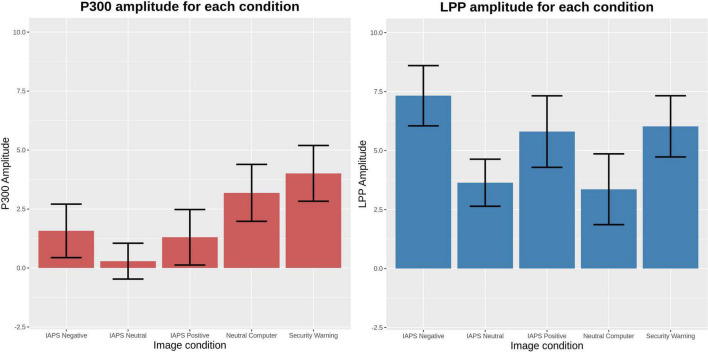
Comparisons of event-related potential mean amplitudes with 95% confidence intervals. These means were assessed using linear mixed effects.

Average amplitudes and confidence intervals of the ERPs are provided in Figure 3. LME comparisons of the 200–600 ms P300 time window revealed that mean amplitudes elicited by the IAPS neutral (β = −3.731; *t* = −4.99; *p* < 0.001), IAPS positive (β = −2.723; *t* = −3.64; *p* < 0.001), or IAPS negative (β = −2.441; *t* = −3.27; *p* = 0.001) condition images were lower than the security warning condition, though the neutral computer-related stimuli was not significantly different. LME comparisons of the 600–1,950 ms LPP time window revealed a mean amplitude elicited by the IAPS neutral stimuli (β = −2.390; *t* = −2.69; *p* = 0.007) and neutral computer-related stimuli (β = −2.666; *t* = −3.00; *p* = 0.003) were significantly lower than the security warnings condition, though the mean amplitude elicited by security warnings was not significantly different from the mean amplitudes elicited by the IAPS positive or IAPS negative stimuli. Figure 3 illustrates the mean ERP amplitudes and 95% confidence intervals for each stimuli condition.

**FIGURE 4:**
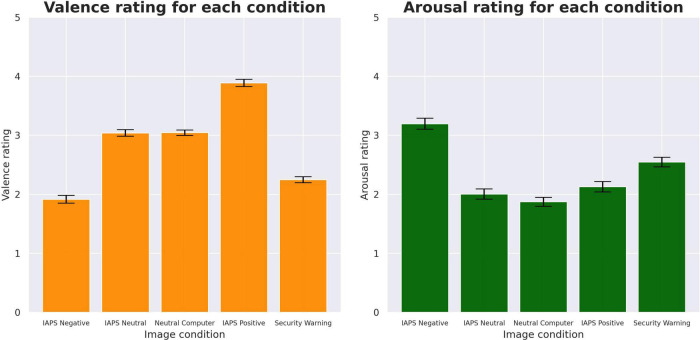
Comparisons of SAM rating mean amplitudes with 95% confidence intervals. These means were assessed using ordinal regression with cumulative link models.

Ordinal regression analysis of the behavioral responses revealed significant differences in both the valence and arousal measures. Comparisons of the valence revealed that negative condition images were rated more negatively than security warnings (β = −0.527; *z* = −8.303; *p* < 0.001) while the IAPS neutral (β = 1.148; *z* = 18.535; *p* < 0.001), computer-related stimuli (β = 1.157; *z* = 18.599; *p* < 0.001), and IAPS positive images (β = 2.351; *z* = 34.204; *p* < 0.001) were rated more positively. Comparisons of the arousal revealed that the IAPS positive (β = −0.417; *z* = −7.178; *p* < 0.001), IAPS neutral (β = −0.548; *z* = −9.346; *p* < 0.001), and neutral computer-related stimuli (β = −0.652; *z* = −11.060; *p* < 0.001) were rated less arousing than security warning stimuli while the IAPS negative (β = 0.534; *z* = 9.295; *p* < 0.001) were rated as more arousing. Figure 4 illustrates the mean SAM responses and 95% confidence intervals for each stimuli condition.

## Discussion

As predicted, the results revealed significantly greater P300 amplitude for both computer-related stimuli—security warnings and neutral—relative to IAPS pictures. We interpret these results to suggest that the participants saw stimuli that were related to computers as task-related and attended to them. These findings were not surprising given that we informed participants that the experiment concerned security notifications. These findings may have limited use beyond suggesting that participants perceived technology-related stimuli as relevant to the task that they were asked to complete. However, it suggests that the P300 could be a useful measure for comparing perceived relevance of various stimuli in future studies involving cognitive processes in information technology, such as habituation (Vance et al., 2014).

The comparisons of amplitudes in the 600–1,950 ms window corresponding to the LPP yielded different insights. Cybersecurity warnings elicited LPPs of comparable amplitude to standardized IAPS stimuli known to elicit emotionally valenced responses, and greater than the LPPs elicited by either neutral IAPS pictures, or neutral computer-related stimuli. This pattern of LPP responses supports our hypothesis that security notification stimuli elicited emotional responses in our participants. This interpretation is corroborated by the finding that on the SAM, participants responded with greater negative valence, and greater arousal, to images of security warnings than to neutral computer-related images, or to positive or neutral IAPS images.

These findings have clear implications for research in cybersecurity behavior. Emotional reactions to security warnings are known to impact decision-making about safe browsing behavior (Hanus and Wu, 2016), suggesting that the presence of the LPP could be a correlate of such deliberation, and could potentially be used to assess this process without interrupting the user’s activity to ask them for a subjective rating of their emotional reaction. Participants in this study elicited negative valence reactions to cybersecurity warnings, as suggested by their negative valence SAM behavioral response when compared to the participants’ responses to the IAPS neutral or neutral technology (non-security related) conditions. Importantly, stronger emotional reactions to varying stimuli may elicit a stronger LPP signal, and the signal was demonstrated in this study to be linked to a negative emotion behavioral response, which is in turn known to be associated with secure cybersecurity behavior (Vance et al., 2014). Cybersecurity researchers can thus leverage this signal to improve on models that draw from protection motivation theory (Rogers, 1985) by providing an alternative non-subjective measure, or by leveraging the LPP to passively measure emotional responses to cybersecurity stimuli. LPP responses can complement previously investigated signals derived from fMRI, eye tracking or field experiments to give added context about the users’ emotional processing and how this relates to other cognitive factors in their decision-making process (Vance et al., 2018).

The findings also have applications to broader research in the role of cognitive processes in information technology use. EEG measures of affective responses can supplement psychological measures and provide additional information about unconscious processing (Dimoka et al., 2011), which can help explain subtle differences in responses to individual stimuli. Research by Bublatzky and Schupp (2012) found that the LPP may distinguish emotional responses independent of paradigm manipulation, suggesting that the LPP could be a more reliable measure of affective responses than many behavioral tasks. The measure could thus be used to give new insight into factors that facilitate safe browsing behavior, as well as insights into whether emotional responses to other warnings encourage desirable action.

There are some limitations to our findings. First, while IAPS stimuli have been extensively tested and standardized, the computer-related stimuli used in this study have possible confounds, such as text and color. Though we controlled for luminance effects, the salience of an image is known to impact the LPP response. For example, Minnix et al. (2013) observed the LPP in the context of advertising and found that emotional and cigarette stimuli elicited larger LPP responses even among non-smokers, suggesting that the stimuli which stand out from others can elicit the LPP. It is possible that the presence of text in the images, the frequency of particular colors (e.g., red and white, common in notifications), or presence of familiar product (e.g., Apple vs. Windows 7) could confound the signal. Future work should consider other textual images and reading ability of the participants when assessing reactions to notifications.

Additionally, this study was limited in the sense that participants were fully informed of the purpose of the study in the consent process. We believe this is what best explains the observed elevation in P300 amplitude elicited by stimuli from the two computer-related conditions. Future work should consider withholding the purpose of the study from participants until the debriefing stage.

Finally, this study required participants to regularly respond with behavioral responses to the stimuli following their presentation. It is possible that the signals observed in this study were influenced by the expectation of a behavioral response. Future work should consider replicating these findings without the requirement of a behavioral response.

We suggest two directions for future work. First, it is possible to apply these findings to create an index of normative affective responses to cybsersecurity notifications, similarly to the work undertaken in the development of the IAPS (Lang, 2005). By providing a comprehensive index of responses to cybersecurity notifications, researchers would be able to identify which designs elicit strong emotional responses. They could then use this index to study behavior in real-world contexts. Doing this would respond to calls by cybersecurity researchers to study more complex cognitive processes, such as the role of affective processing when making decisions about computer safety (Anderson et al., 2016; Vance et al., 2018).

Second, it is possible to apply the measure outside of the context of cybersecurity notifications and into other stimuli of interest, such as social media or other notification contexts. A past study by Marín-Morales et al. (2018) applied machine learning to detect EEG signals elicited by affective responses in a virtual reality environment in a paradigm that leveraged IAPS pictures. This suggests that the LPP signal could be applicable in applied, more ecologically valid contexts. Future research could apply such techniques to observe automatic affective processing in novel contexts, such as in interactive experiments with internet browsing behavior or field research.

## Conclusion

In a world where information technology plays an increasingly important role in our lives, it is critical that we discover ways of using it safely. It is critical for computing companies to understand users’ decision-making processes when using computers, so that they can identify the best ways to encourage safe behavior. In this paper, we described a potential marker of affective processing which has been posited to play a role in users’ decision process in the context of cybersecurity notifications. We observed increased amplitude in the late-positive-potential (LPP) among responses to images of cybersecurity notifications when compared to both standardized, emotionally neutral images and neutral (non-security related) computer images. Researchers may benefit by leveraging these signals to give additional insights into the role of emotional processing when making decisions about safe computer use.

## Data Availability Statement

The datasets presented in this study can be found in online repositories. The names of the repository/repositories and accession number(s) can be found below: https://doi.org/10.5683/SP3/RDXI6P.

## Ethics statement

The studies involving human participants were reviewed and approved by the Dalhousie University Research Ethics Board. The patients/participants provided their written informed consent to participate in this study.

## Author contributions

CC, JA, and AN designed the study and wrote the final manuscript. JH collected the data. CC, JH, and AN contributed to the analysis of the data. All authors contributed to the article and approved the submitted version.

## Conflict of Interest

The authors declare that the research was conducted in the absence of any commercial or financial relationships that could be construed as a potential conflict of interest.

## Publisher’s Note

All claims expressed in this article are solely those of the authors and do not necessarily represent those of their affiliated organizations, or those of the publisher, the editors and the reviewers. Any product that may be evaluated in this article, or claim that may be made by its manufacturer, is not guaranteed or endorsed by the publisher.

## References

[B1] Anderson B. B. Vance A. Kirwan C. B. Eargle D. Jenkins J. L. (2016). How users perceive and respond to security messages: A NeuroIS research agenda and empirical study. *Eur. J. Inform. Syst.* 25 364–390. 10.1057/ejis.2015.21

[B2] Bradley M. M. Hamby S. Löw A. Lang P. J. (2007). Brain potentials in perception: picture complexity and emotional arousal. *Psychophysiology* 44 364–373. 10.1111/j.1469-8986.2007.00520.x 17433095

[B3] Bradley M. M. Lang P. J. (1994). Measuring emotion: the self-assessment manikin and the semantic differential. *J. Behav. Ther. Exp. Psychiat.* 25 49–59. 10.1016/0005-7916(94)90063-9 7962581

[B4] Brown S. van Steenbergen H. Band G. de Rover M. Nieuwenhuis S. (2012). Functional significance of the emotion-related late positive potential. *Front. Hum. Neurosci.* 6:33. 10.3389/fnhum.2012.00033 22375117 PMC3287021

[B5] Bublatzky F. Schupp H. T. (2012). Pictures cueing threat: brain dynamics in viewing explicitly instructed danger cues. *Soc. Cogn. Affect. Neurosci.* 7 611–622. 10.1093/scan/nsr032 21719425 PMC3427861

[B6] Christensen R. H. B. (2018). *Cumulative Link Models for Ordinal Regression with the R Package Ordinal.* Available online at: http://cran.uni-muenster.de/web/packages/ordinal/vignettes/clm_article.pdf (accessed April 2, 2022).

[B7] Conrad C. Aziz J. Smith N. Newman A. (2020). “What Do Users Feel? Towards Affective EEG Correlates of Cybersecurity Notifications,” in *Information Systems and Neuroscience*, eds Davis F. D. Riedl R. vom Brocke J. Léger P.-M. Randolph A. B. Fischer T. (Cham: Springer International Publishing), 153–162. 10.1007/978-3-030-60073-0_17

[B8] Davidson D. J. (2009). Functional Mixed-Effect Models for Electrophysiological Responses. *Neurophysiology* 41 71–79. 10.1007/s11062-009-9079-y

[B9] Delorme A. Makeig S. (2004). EEGLAB: an open source toolbox for analysis of single-trial EEG dynamics including independent component analysis. *J. Neurosci. Methods* 134 9–21. 10.1016/j.jneumeth.2003.10.009 15102499

[B10] Dimoka A. Pavlou P. A. Davis F. D. (2011). Research Commentary: NeuroIS: The Potential of Cognitive Neuroscience for Information Systems Research. *Inform. Syst. Res.* 22 687–702.

[B11] Garcia-Garcia J. M. Penichet V. M. Lozano M. D. (2017). *Emotion Detection: A Technology Review.* Cancun, Mexico: ACM Press, 1–8. 10.1145/3123818.3123852

[B12] Gerger G. Leder H. Kremer A. (2014). Context effects on emotional and aesthetic evaluations of artworks and IAPS pictures. *Acta Psychol.* 151 174–183. 10.1016/j.actpsy.2014.06.008 24983515

[B13] Gramfort A. Luessi M. Larson E. Engemann D. A. Strohmeier D. Brodbeck C. (2014). MNE software for processing MEG and EEG data. *NeuroImage* 86 446–460. 10.1016/j.neuroimage.2013.10.027 24161808 PMC3930851

[B14] Guo K. H. Yuan Y. Archer N. P. Connelly C. E. (2011). Understanding Nonmalicious Security Violations in the Workplace: A Composite Behavior Model. *J. Manage. Inform. Syst.* 28 203–236. 10.2753/MIS0742-1222280208

[B15] Hajcak G. Olvet D. M. (2008). The persistence of attention to emotion: brain potentials during and after picture presentation. *Emotion* 8 250–255. 10.1037/1528-3542.8.2.250 18410198

[B16] Hanus B. Wu Y. (2016). Impact of Users’ Security Awareness on Desktop Security Behavior: A Protection Motivation Theory Perspective. *Inform. Syst. Manage.* 33 2–16. 10.1080/10580530.2015.1117842

[B17] Johnston A. C. Warkentin M. (2010). Fear Appeals and Information Security Behaviors: An Empirical Study. *MIS Quart.* 34 549–566. 10.2307/25750691

[B18] Landowska A. Miler J. (2016). “Limitations of emotion recognition in software user experience evaluation context,” in *2016 Federated Conference on Computer Science and Information Systems (FedCSIS)*, (Poland: IEEE), 1631–1640.

[B19] Lang P. J. (2005). *International Affective Picture System (IAPS): Affective Ratings of Pictures and Instruction Manual. Technical Report A-8.* Gainesville, FL: NIMH, Center for the Study of Emotion & Attention.

[B20] Leventon J. S. Bauer P. J. (2016). Emotion regulation during the encoding of emotional stimuli: effects on subsequent memory. *J. Exp. Child Psychol.* 142 312–333. 10.1016/j.jecp.2015.09.024 26597138

[B21] Luck S. J. (2014). *An Introduction to the Event-Related Potential Technique*, second Edn. Cambridge: MIT Press.

[B22] Marín-Morales J. Higuera-Trujillo J. L. Greco A. Guixeres J. Llinares C. Scilingo E. P. (2018). Affective computing in virtual reality: emotion recognition from brain and heartbeat dynamics using wearable sensors. *Sci. Rep.* 8:13657. 10.1038/s41598-018-32063-4 30209261 PMC6135750

[B23] Minnix J. A. Versace F. Robinson J. D. Lam C. Y. Engelmann J. M. Cui Y. (2013). The late positive potential (LPP) in response to varying types of emotional and cigarette stimuli in smokers: a content comparison. *Int. J. Psychophysiol.* 89 18–25. 10.1016/j.ijpsycho.2013.04.019 23643564 PMC3771859

[B24] Pierce J. (2009). Generating stimuli for neuroscience using PsychoPy. *Front. Neuroinform.* 2:10. 10.3389/neuro.11.010.2008 19198666 PMC2636899

[B25] Rogers R. W. (1985). Attitude Change and Information Integration in Fear Appeals. *Psychol. Rep.* 56 179–182. 10.2466/pr0.1985.56.1.179

[B26] Samtani S. Kantarcioglu M. Chen H. (2020). Trailblazing the artificial intelligence for cybersecurity discipline: a multi-disciplinary research roadmap. *ACM Trans. Manage. Inform. Syst.* 11 1–19. 10.1145/3430360

[B27] Schaumont P. Montuschi P. (2021). Computer Security at the Forefront of Emerging Topics in Computing. *Computer* 54 4–5. 10.1109/MC.2021.3084474

[B28] Skiendziel T. Rösch A. G. Schultheiss O. C. (2019). Assessing the convergent validity between the automated emotion recognition software Noldus FaceReader 7 and Facial Action Coding System Scoring. *PLoS One* 14:e0223905. 10.1371/journal.pone.0223905 31622426 PMC6797095

[B29] Tremblay A. Newman A. J. (2015). Modeling nonlinear relationships in ERP data using mixed-effects regression with R examples. *Psychophysiology* 52 124–139. 10.1111/psyp.12299 25132114

[B30] van Bavel R. Rodríguez-Priego N. Vila J. Briggs P. (2019). Using protection motivation theory in the design of nudges to improve online security behavior. *Int. J. Hum. Comp. Stud.* 123 29–39. 10.1016/j.ijhcs.2018.11.003

[B31] Vance A. Anderson B. Kirwan C. B. Eargle D. (2014). Using Measures of Risk Perception to Predict Information Security Behavior: Insights from Electroencephalography (EEG). *J. Assoc. Inform. Syst.* 15 679–722. 10.17705/1jais.00375

[B32] Vance A. Jenkins J. L. Anderson B. B. Bjornn D. K. Kirwan C. B. (2018). Tuning Out Security Warnings: A Longitudinal Examination of Habituation Through fMRI, Eye Tracking, and Field Experiments. *MIS Quart.* 42 355–380. 10.25300/MISQ/2018/14124

[B33] Vogel E. K. Luck S. J. (2002). Delayed working memory consolidation during the attentional blink. *Psychonom. Bull. Rev.* 9 739–743. 10.3758/BF03196329 12613677

